# A systematic review: polyphenol’s effect on food allergy via microbiome modulation

**DOI:** 10.3389/fmicb.2025.1673472

**Published:** 2025-11-18

**Authors:** Tekan Singh Rana, Rishipal Rastrapal Bansode, Jenny Pakhrin Rana, Leonard L. Williams

**Affiliations:** 1 Center for Excellence in Post-Harvest Technologies, North Carolina Agricultural and Technical State University, Kannapolis, NC, United States; 2 Department of Biology, North Carolina Agricultural and Technical State University, Greensboro, NC, United States

**Keywords:** polyphenol, food allergy, microbiome, 16s rRNA sequencing, egg allergy, milk allergy, soyabean allergy, shrimp allergy

## Abstract

**Introduction:**

Food allergy is an increasing health concern worldwide. Microbes, food allergies, and polyphenols are found to be interrelated. However, studies relating polyphenols’ effect on food allergy via microbiome modulation are scarce, and there is a lack of common signature microbiome modulation patterns. Thus, this review aims to summarize the effect of polyphenols on food allergy via microbiome modulation.

**Methods:**

Research articles were searched from Scopus, PubMed, ScienceDirect, and Web of Science database. The in vivo and in vitro studies were assessed via SYRCLE risk of bias and modified CONSORT checklist, respectively. The population characteristics and experimental details were extracted, and the data were synthesized narratively.

**Results:**

The included studies were free of selective reporting of results. The allergy of egg (ovalbumin), milk (𝛽-lactoglobulin), soybean (𝛽-conglycinin), and shrimp allergy contributed to 54%, 23%, 15%, and 8% of the total included studies, respectively. The used compounds were a different source or types of polyphenols such as cocoa, cyanidin-3-O-glucoside (C3G), avenanthramide's (AVA), rosmarinic acid (RA), neohesperidin, and fermented apple juice for egg allergy, luteolin, and green tea polyphenol (GTP) for soybean allergy, and flavonoids (Luteolin, myricetin and hyperoside), ferulic acid, and luteolin for milk allergy. Allergies of milk, egg, wheat, and shrimp occurred with the reduction *of Lactobacillus*, *Alistipes*, *Odaribactor*, *Akkermansia*, *Bacteroides*, and *Lachnospiraceae_NK4A136_group* and an increase of *Prevotella*, *Alloprevotella*, *Faecalibaculum*, *Helicobactor*, *Blautia*, *Clostridium*, and *Staphylococcus.* The polyphenols modulated these microbes in order to attenuate the food allergies.

**Discussion:**

The types of polyphenols, food allergies, animal model used, and taxonomic resolution of the microbiome studies lead to variation in the results. Thus, by increasing the studies on effect of polyphenols on individual food allergies, and combining with higher taxonomic resolution techniques such as shotgun metagenomics along with metabolomics would increase reliability of the results of the future studies.

## 1 Introduction

Food allergy is a serious health concern (Camps-Bossacoma et al., 2017; Yang et al., 2023) and about 5% of adults and 8% of children suffer food allergy worldwide (Li et al., 2022; Loh and Tang, 2018). Studies show the relationship among microbiota, polyphenolic compounds, and food allergies (Camps-Bossacoma et al., 2017; Yang et al., 2023; Li et al., 2022; Liu et al., 2023; Wang et al., 2022; Zhou et al., 2023) or cytokine-induced inflammations (Liu et al., 2021). The commensal gut microbes help the breakdown of dietary foods, produce short-chain fatty acid (SCFA), protect intestinal epithelial cells (IEC), modulate protective barriers, promote mucosal immunity system by developing tolerogenic CD103^+^ dendritic cells (DC) which influence regulatory T cells (T-reg) and IgA production from B cell, and metabolites production (Chen et al., 2016; Goldberg et al., 2020; Gu et al., 2022; Kourosh et al., 2018).

**Figure 1:**
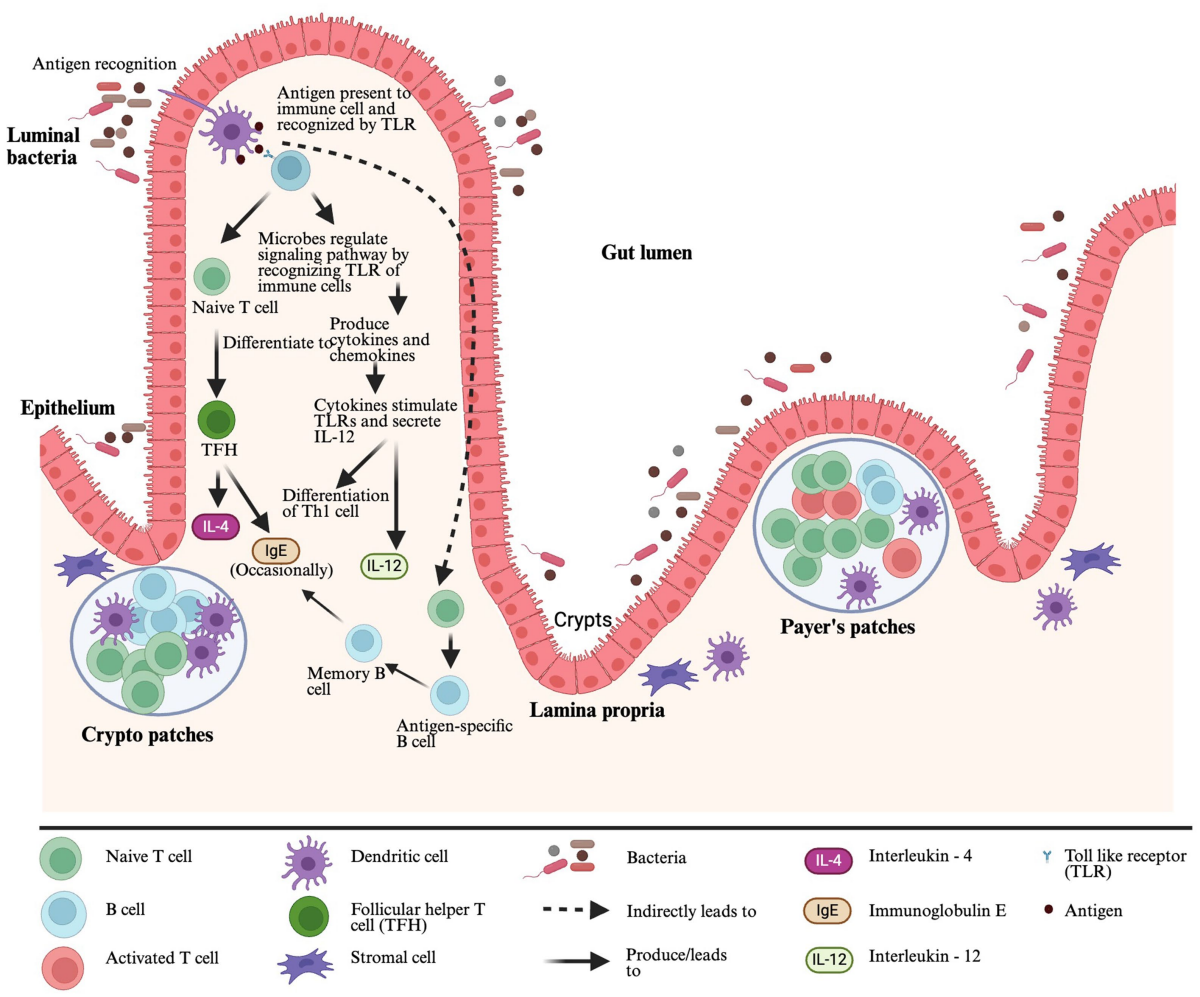
Gut-associated lymphoid tissue and microbe interaction.

Gut-associated lymphoid tissue (GALT) is a mucosal immune system in the gut and the largest lymphoid tissue in the human body. It helps to promote oral tolerance to specific food allergens by distinguishing allergic and non-allergic food antigens (Ganesh and Versalovic, 2015). The GALT comprises of lymphoid structures and scattered lymphocytes with specialized functions (e.g., Natural killer cells, B cells, and T cells) in epithelium and lamina propria (LP). The lymphoid structures include Payer’s patches (PPs), crypto patches (CPs), DC, stromal cells around the crypts of the small intestine, intraepithelial lymphocytes (IELs), intestinal epithelial cells (IECs) and mesenteric lymph nodes (MLNs) (Sajdel-Sulkowska and Sajdel-Sulkowska, 2019) (Figure 1). The DC is one of the most important antigen-presenting cells (APC), and after getting antigens from the gut lumen, DCs present them to toll-like receptors (TLRs). The TLRs are pattern recognition receptors expressed in the immune cells and recognized by gut microbiota. After recognition of TLRs, microbes can regulate signaling pathways to communicate with a host producing pro- or anti-inflammatory cytokines and chemokines (Liu et al., 2022). When cytokines stimulate TLRs, they secrete Interleukin 12 (IL-12), and IL-12 helps in Type 1 helper CD4^+^ T cell differentiation in the presence of antigens (Ganesh and Versalovic, 2015). After interacting with antigen-containing DCs, naïve CD4^+^ T cells differentiate into T follicular helper (TFH) cells. These TFH cells produce IL-4 in response to allergens, and they can also occasionally generate Immunoglobulin E (IgE), replacing the role of Th2 cells in generating IgE (Hong et al., 2019). The DCs present antigens with T cells and can share that information with B cells separately. In that case, naïve B cells will turn into antigen-specific B cells, further differentiating into memory B cells or ultimately turning into plasma cells that produce IgE specific to an antigen (Shi et al., 2015) (Figure 1).

Polyphenol’s inhibitory or stimulatory effects on microbes depend upon the polyphenol’s structure and bacterial species/strains (Makarewicz et al., 2021). Polyphenols have antimicrobial properties with various mechanisms. Polyphenols interact with bacterial proteins on the cell wall, cell membrane, and with those proteins involved in the fundamental metabolism, inhibit DNA synthesis or cause DNA cleavage, disturb membrane permeability, antibiotic resistance, and enzyme formation. Moreover, polyphenols also inhibit ATP synthase and ATPase function, biofilm formation, and quorum sensing activities (Makarewicz et al., 2021; Rodríguez-Daza et al., 2021; Ashwin et al., 2021; Plamada and Vodnar, 2021).

**Figure 2:**
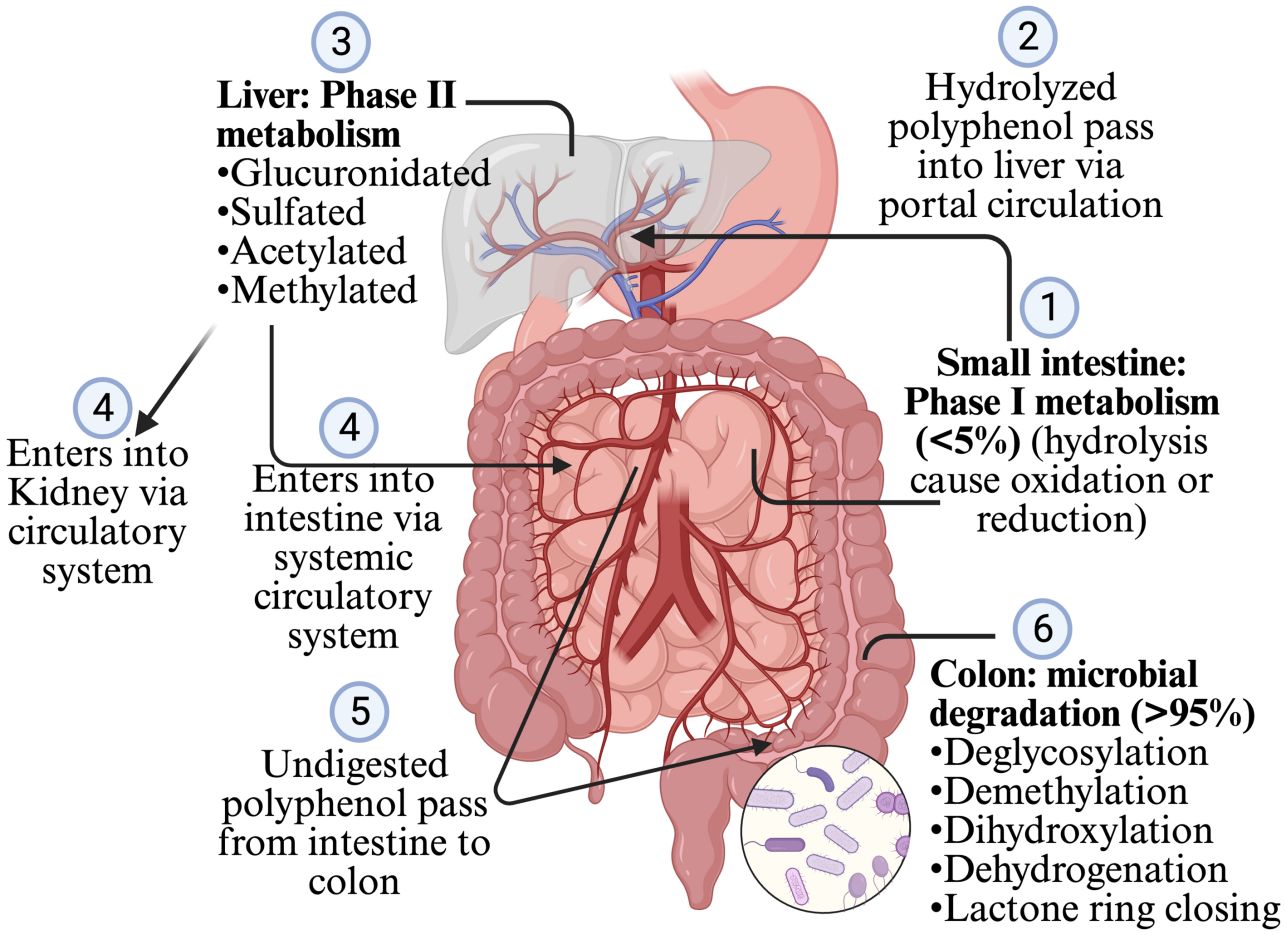
Digestion and metabolism of polyphenols.

Less than 5% of polyphenols consumed are absorbed in the stomach and intestine, and >95% are undigested and reach the colon and interact with gut microbiota (Makarewicz et al., 2021; Rodríguez-Daza et al., 2021; Ashwin et al., 2021; Loo et al., 2020; Rowland et al., 2018). Most of the polyphenols are found as glycosides or in the polymers, so they need to be converted into aglycone and simple compounds so that enterocytes can absorb them. However, some glycosides, such as anthocyanins, can be absorbed without processing (Ashwin et al., 2021; Plamada and Vodnar, 2021; Loo et al., 2020; Rowland et al., 2018). The polymerization, and types of polyphenols such as flavonoids and non-flavonoids, affect the microbial conversion of the polyphenols. Flavonoids are composed of two benzene rings (A and B rings) linked to a heterocyclic pyrone C-ring. Simple phenolics derived from the A and B rings are released after the gut microbiota breaks down the C-ring in different positions. The hydroxylation pattern and the position of the B-ring determines the types of resulting phenotypes (Plamada and Vodnar, 2021; Ozdal et al., 2016). After absorption from the intestine, the polyphenol is mildly oxidized or reduced by hydrolysis (phage I metabolism, which increases the polarity of polyphenols) when it passes through enterocytes (Figure 2). The resulting simpler forms of polyphenols are transferred to the liver via the portal circulation, where they are glucuronidated, sulfated, acetylated or methylated (phage II metabolism that adds the chemical radicals into polyphenol) (Ashwin et al., 2021; Plamada and Vodnar, 2021; Rowland et al., 2018; Mithul Aravind et al., 2021). The resulting metabolites after phage II metabolism enter different organs via the systemic circulatory system. The undigested polyphenols in the intestine pass into the colon and are further metabolized into simpler forms by GM. The processes involved during this transformation are deglycosylation, demethylation, dihydroxylation, dehydrogenation, and closing of the lactone ring in the lower colon (Ashwin et al., 2021; Plamada and Vodnar, 2021; Rowland et al., 2018). After the resulting metabolites or polyphenols are absorbed from the colon, they go to phase II metabolism in the intestinal tissue and liver. Enterohepatic circulation helps to excrete the conjugated compounds back to the gut, which are again deconjugated by microbes and reabsorbed (Plamada and Vodnar, 2021; Loo et al., 2020) (Figure 2). The Daidzein, ellagitannins, lignans, proanthocyanidins converts primarily (80–90%) into O-desmethylangolensin or S-equol (30–50%), urolithins, enterolactones (and ultimately into enterodiol in human), and isomers of valerolactones (ultimately into phenolic acid), respectively. Similarly, isoflavone converts into propanoic acid or equol, anthocyanin into phenolic acid or phloroglucinol acid, quercetin into hippuric acid or benzoic acid, neochlorogenic acid into caffeic acid and quinoic acid, trans-resveratrol into piceid and resveratrolozide, and curcumin into ferulic acid and dihyroferulic acid (Makarewicz et al., 2021; Rodríguez-Daza et al., 2021; Plamada and Vodnar, 2021; Rowland et al., 2018; Ozdal et al., 2016; Mithul Aravind et al., 2021). The resulting polyphenol metabolites are more bioactive (bioaccessible and absorbed) than the parent polyphenol (Rowland et al., 2018).

Although studies had proven the association between food allergy and microbiome (Bunyavanich and Berin, 2019; Iweala and Nagler, 2023; Nance et al., 2020; Zhao et al., 2020), polyphenol and microbiome (Catalkaya et al., 2020; De Rossi et al., 2025; Kumar Singh et al., 2019; Piekarska-Radzik and Klewicka, 2020), and polyphenol and allergies (Zeng et al., 2022; Wu et al., 2018; Zhang et al., 2024; Wu et al., 2023), studies investigating the effect of polyphenol on food allergies via microbiome modulation are scarce and are in the initial stage (Li et al., 2022). Thus, this systematic review aims to determine common microbiome modulation pattern of polyphenols to mitigate food allergy.

## 2 Methodology

**Figure 3:**
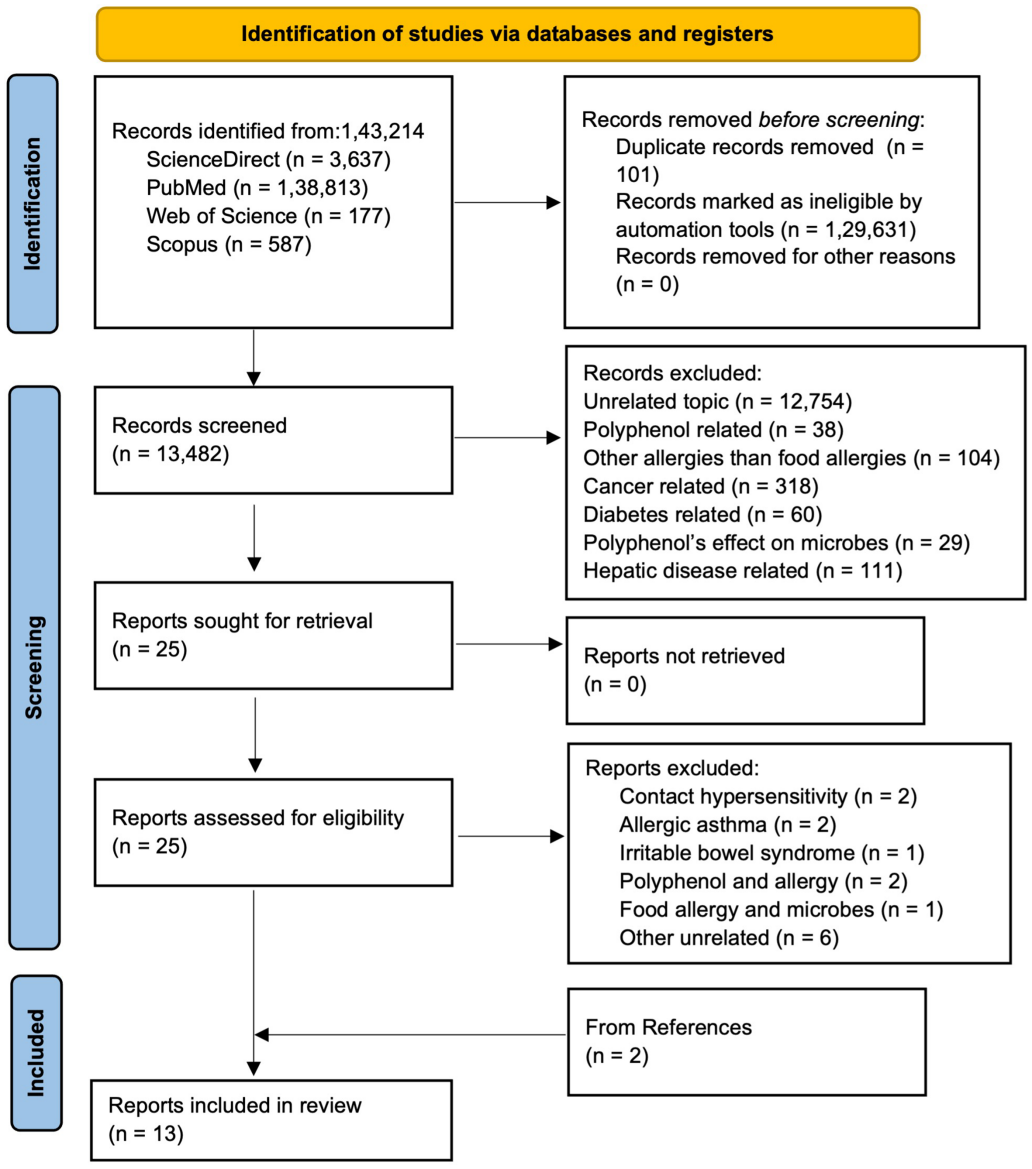
PRISMA flow diagram showing literature search and selection process.

The Preferred Reporting Items for Systematic Reviews and Meta-analysis (PRISMA) guideline was followed to conduct the systematic review (Page et al., 2021). Articles published until 2025, were searched from various databases: ScienceDirect, PubMed, Web of Science, and Scopus. The articles were searched in different databases using following combination of keywords: (a)In Scopus and Web of Science, (“hypersensi*” OR “food allerg*” OR “allerg*” OR “anaphyla*”) AND (“microbio*” OR “microorga*”) AND (“proanthocya*” OR “anthocya*” OR “procyan*"OR “flav*” OR “polypheno*”); (b) In ScienceDirect, (“hypersensitivity” OR “food allergy” OR “anaphylaxis”) AND (“microbiome” OR “microorganism”) AND (“procyanidins”OR “flavonoids” OR “polyphenols”); and (c) In PubMed, ((((((“Food Hypersensitivity”[Mesh]) OR “Hypersensitivity”[Mesh]) AND “Gastrointestinal Microbiome”[Mesh]) OR “Microbiota”[Mesh]) AND “Polyphenols”[Mesh]) OR “Flavonoids”[Mesh]) OR “Proanthocyanidins”[Mesh]. As shown in PRISMA flow diagram (Figure 3), from a total of 1,43,214 articles obtained using aforementioned keywords from different databases, 101 were removed in deduplication, 1,29,631 were removed using automation process by search tools as they were one of the following categories: other than original research articles, and published in other than English. Out of 13,482 remaining screened articles, 13,457 were excluded during title and abstract screening. Out of the 25 remaining articles, two were related to contact hypersensitivity, two articles were related allergic asthma, one was related to irritable bowel syndrome, two were related to polyphenol and allergy, one was related to food allergy and microbes only, and six were other unrelated articles. Thus, 11 articles were obtained from screening and two articles were obtained from reference and citation of the 11 articles leading to 13 total articles for this systematic review. Two authors (TR and JR) agreed upon search criteria, searched and screened articles, and discussed and resolved disagreements with the third author (RB). Three authors (TR, RB, and JR) independently extracted data on objectives, tissue or sample type, treatment, treatment administration route, treatment dose, and duration, sequencing type, and microbiome change, and population characteristics (animal type, age, weight, sex, total number). The evidence in articles was determined with the PICO framework as follows: population: animal (mice, rats, pigs) and human, intervention: polyphenol, comparison: food allergen, output: change in microbiome.

Three authors (TR, RB, and JR) performed the risk of bias. The risk of bias evaluation of *in vivo* studies was performed according to SYRCLE’s risk of bias tool (Hooijmans et al., 2014). Comprehension and unbiases on abstract, background and rationale, objectives, hypothesis, intervention, outcome, statistical method, outcome, limitation, and funding were evaluated. For *in vitro* studies, the risk of bias was calculated based on a modified CONSORT checklist (Faggion, 2012; Lam et al., 2024). The process of complete randomization, blinding, unbiased and complete reporting of the articles was evaluated. Due to differences in treatment, objective and overall design, and outcomes among selected articles, we narratively synthesized the articles. The authors agreed upon the synthesis process.

## 3 Results

### 3.1 Risk of bias

The *in vitro* studies did report background, objectives, outcomes, and limitations. However, they did not mention the hypothesis, and one of the studies did not clearly mention the statistical method used to analyse the data (Supplementary Table 1). All the *in vivo* studies did not explain the study’s randomization process and blinding steps. Two of the studies did not mention reasons for incomplete outcomes. All of them were free from selective outcome reporting (Supplementary Table 2).

### 3.2 Population/study characteristics

Two studies were done on 3-week-old Lewis and Brown Norway rats, seven on 5–8 weeks-old BALB/c mice, one on C57BL/6 J mice, one on 18-day-old piglets, and two on incubation of human fecal matter in the artificial chamber (Table 1). All animal studies used either female or male animals. The allergy on ovalbumin, beta-lactoglobulin (milk) and soybean, and shrimp contributed 54, 23, 15, and 8% of the included studies, respectively (Table 1).

**Table 1:** Study/population characteristics.

Ref	Animal/Cell	Animal type	Age	Weight (g)	Sex	Total number	Number used for microbiome analysis	Allergy Type
Wang et al. (2022)	Human/invitro	NA	NA	NA	NA	3	3	BLG
Liu et al. (2021)	Human/invitro	NA	18–22 years		Male + Female	3	3	BLG
Yang et al. (2023)	Mice	BALB/c	5 weeks	18–20	Female	40	40	OVA
Liu et al. (2023)	Mice	BALB/c	6 weeks	NA	Male	80	40	OVA
Li et al. (2022)	Mice	BALB/c	6–8 weeks	NA	Male	30	30	OVA
Camps-Bossacoma et al. (2017)	Rat	Lewis	3 weeks	NA	Female	18	9	OVA
Zhou et al. (2023)	Rat	Brown Norway	3 weeks	39.15+ to 4.38	Female	18	18	Soyabean
Liang et al. (2024)	Piglets	Crossbreed (Duroc X Landrace X Large white)	18 days	NA	NA	18	18	Soyabean
Feng et al. (2024)	Mice	BALB/c	6 weeks	NA	Female	48	NA	Shrimp
Liu et al. (2025)	Mice	BALB/c	5–6 weeks	NA	Female	30	30	OVA
Ma et al. (2025b)	Mice	BALB/c	6 weeks	18–22	Female	NA	NA	OVA
Ma et al. (2025a)	Mice	C57BL/6 J mice	8 weeks	NA	Male + Female	NA	18	OVA
Wang et al. (2024)	Mice	BALB/c	5–6 weeks	NA	Female	60	60	BLG

NA, Not available, BLG, 𝛽-lactoglobulin, OVA, ovalbumin.

### 3.3 Microbiome and food allergies

#### 3.3.1 Egg allergies

In general, ovalbumin (OVA) seem to reduce *Lactobacillus* (Yang et al., 2023; Li et al., 2022; Ma et al., 2025b; Liu et al., 2025), *Alistipes* (Li et al., 2022; Ma et al., 2025b), *Prevotella* (Yang et al., 2023), *Akkermansia* (Camps-Bossacoma et al., 2017), and members of lachnospiraceae such as *Unclassified_f_lachnospiraceae*, and *Lachnospiraceae_NK4A136_group* (Ma et al., 2025b; Liu et al., 2025). The OVA promoted the *Bacteroides* (Camps-Bossacoma et al., 2017; Ma et al., 2025b), *Helicobactor* (Li et al., 2022), *Faecalibaculum* (Liu et al., 2025), and *Alloprevotella* (Ma et al., 2025b). At higher level of classification, OVA promotes muribaculaceae (Yang et al., 2023; Liu et al., 2025), campylobacteria (Li et al., 2022), and proteobacteria (Ma et al., 2025a), meanwhile the OVA reduced firmicutes (Li et al., 2022; Ma et al., 2025a, 2025b; Liu et al., 2025). Use of various polyphenol helped to alleviate allergy via reducing the microbes promoted by OVA and promoting those microbes reduced by OVA. However, due to variation on the polyphenol and animal model used, diverse effect of polyphenol were found on microbes. For example, cocoa diet promoted *Lactobacillus*, *Provotella*, *Anastipes*, and it reduced *Clostridium* and *Blautia*. On the other hand, cyanidin-3-O-Glucoside promoted *Rosburia*, *Blautia*, and *Lachnospiraceae_NK4A136_group* (Table 2).

**Table 2:** Main findings of the egg allergy studies.

Ref	Objectives	Tissue/cells/sample	Collection time	Treatment; control	Administration route	Treatment dose	Treatment duration	Sequencing type	↑↓ Microbiome
Camps-Bossacoma et al. (2017)	Influence of oral sensitization on gut microbiota	Fecal drops	before sensitization and once/week. For metagenomics, on day 28	OVA 50 mg + cholera toxin 30 μg in 1 mL D. I. H_2_O	Oral	10% cocoa diet + OVA + cholera toxin; Standard diet + OVA 50 mg + cholera toxin 30 μg in 1 mL D. I. H_2_O; Standard diet + 1 mL vehicle	Three times/week for 3 weeks	16S metagenomics	OVA ↓ *Clostridium metallolevans*, *Allobaculum* sp., ↓↓ *Staphylococcus equorum*, *Akkermansia muciniphila.* OVA *↑↑ Bacillus*, *Christensenella*, and *Anaeroplasma* compared to standard food (SF). OVA ↓↓ *Bacteroides uniformis* and *Prevotella* sp., but were present in OVA/C and SF. OVA/C ↓ *Allobaculum* sp., *Holdemania* sp. *and ↑ Clostridium metallovans* compared to SF. OVA *↓ Ruminococcus flavefaciens* and ↑ *Bacteroides uniformis* compared to OVA, ↑ *Lactobacillus reuteri*, *Prevotella*, and *Anaerostipes* sp. compared to SF and OVA. *Ralstonia* sp., *Desulfovibrio* sp., and *Prevotella copri* were ↑↑ due to OVA/C compared to SF and OVA. The *Clostridium perfringens*, *Blautia producta*, *Epulopiscium* sp., *Coprobacillus* sp., and *Desulfovibrio* sp. *↓↓* compared to SF and OVA. At phylum level, cocoa diet ↓ Firmicutes and Proteobacteria and ↑ Tenericutes and Cyanobacteria.
Li et al. (2022)	Anti-food allergic activity of cyanidin-3-O-glucoside (C3G) delivered by enteric sodium alginate in *in vivo*	a. Serum b. Blood and intestinal tissues c. Feces	a. 1 h after last challenge b. 2 days after last challenge	OVA + C3G, OVA + C3G + LVA; OVA + Alum sensitization + PBS challenge (−ve), OVA + selective histamine H1 receptor antagonist, i.e., Lora (+ve)	a. Sensitization: Intraperitoneal (IP) injection. b. Challenge: Gavage feeding	a. Sensitization: Twice with OVA 100 μg + alum 2 mg in 200 μL PBS b. Challenge: OVA 50 mg OVA in 200 μL PBS. LVA + C3G (3:1 mass ratio). C3G 25 mg/kg bwt, Lora 20 mg/kg bwt	a. Sensitization: Day 0 and day 14 b. Challenge: Day 28 to Day 40 at 3 days interval	16S rRNA gene amplicon sequencing	At phylum level, the OVA ↓ Bacteroidota and Firmicutes and ↑ Campilobacterota. The Lora and C3G ↑ relative abundance of Bacteroidota and Firmicutes and ↓ Campilobacterota. At genus level, OVA ↑ *Helicobacter* and *Turicibacter* and ↓ *Lactobacillus* and *Alistipes.* LVA + C3G had opposite effect than that of OVA treatment on these bacteria. LVA + C3G also ↑ *Odaribacter*.
Liu et al. (2023)	Avenanthramide’s (AVA) effect on colonic damage induced by food allergy and its mechanism	a. Blood b. colon tissue c. feces from colon	After 38 days	In experiment 1, OVA + LAVA or OVA + HAVA; OVA (+ve) or saline (−ve) In experiment 2, OVA + HAVA, OVA + APO, OVA + HAVA + APO; OVA	In Experiment 1 and 2, a. Sensitization: IP injection. b. Callenge: Intragastrically. c. AVA and saline intragastricllay and APO IP injection	In Experiment 1 and 2, a. Sensitization: Alum 1 mg + OVA 50 μg in 200 μL saline on day 0 and 14 b. Challenge: OVA 50 mg every other day (Six times from day 28 to 38). AVA were administred after 1 h of OVA treatment in sensitization and challenge day. In Experiment 2, HAVA 20 mg/kg bwt and LAVA 10 mg/kg bwt daily. APO 4 mg/kg bwt in 20 μL DMSO every other day 1 h before sensitization and challenge	38 days	16S rRNA gene sequencing	AVE ↓ propionate -producing bacteria Muribaculaceae and ↑ butyrate producing bacteria such as *Roseburia*, *Blautia*, and *Lachnospiraceae_NK4A136_group*.
Yang et al. (2023)	Anti-allergic activity and mechanism of rosmarinic acid (RA) in OVA-induced allergy in mice	a. Serum b. Spleen c. Jejunum d. Feces	After last challenge on day 40	OVA + RA-low, OVA + RA-mid, OVA + RA-high; OVA (+ve), no OVA (−ve)	a. Sensitization: IP injection b. Challenge: Oral. RA via oral gavage	a. Sensitization: Ova 5 mg/kg bwt + Alum 2 mg in 200 μL PBS b. Challenge: OVA 2.5 g/kg bwt in PBS RA-low 30 mg/kg, RA-mid 90 mg/kg, and RA-high 270 mg/kg	a. Sensitization: day 0 and 14. b. Challenge: from day 28, in every 3 days. RA once a day from day 27 to 40	16S rRNA gene sequencing	At phylum level, compred to -ve control, all treatments and +ve control ↓ Bacteroidetes and Firmicutes. OVA ↑ Firmicutes/Bacteroidetes ratio. RA ↓Firmicutes/Bacteroidetes ratio casued by OVA. At genus level, compared to -ve control, OVA ↓ *Lactobacillus* and *Prevotella* and ↑ Muribaculaceae and RA slightly ↓ Muribaculaceae.
Liu et al. (2025)	Anti-allergic effect of neohesperidin dihydrochalcone (NHDC) and neohesperidin (NH)	a. Feces b. Jejunum c. Spleen d. Mesenteric lymphnode	After sacrifice on day 58	OVA + NHDC, OVA + NH, OVA + CT; OVA(+ve), PBS (−ve)	a. Sensitization: IP injection b. Challenge: Oral gavage	a. Sensitization: control group with 200 μL of PBS. Food allergy model and other treatment with 100 μg OVA with 2 mg alum b. Challenge: 50 mg OVA in 200 μL PBS	a. Sensitization: Days 0 and 14. b. Challenge: from day 28 to 58 in every 3 days interval. The NHDC, NH, and CT daily from day 1.	16rRNA gene sequencing	At phylum level, food allery ↓ Firmicutes/Bacteroidetes ratio. At genus level, allergy also ↑ Muribaculaceae, *Faecalibaculum* and ↓ *Lactobacillus* and Lachnospiraceae. Compared to NH, NHDC has ↑ abudance of *Lactobacillus*, Lachnospiraceae, and *Akkermansia.*
Ma et al. (2025b)	Modulatory effect of fermented apple juice (FAJ) on food allergy	a. Feces b. Serum c. Jejunum	After 34 days	OVA + FAJ; OVA (+ve), no OVA/ FAJ (−ve)	Sensitization and challenge: oral	a. Sensitization: OVA 1 mg + cholera toxin 10 μg. FAJ 0.15 mL/10 g mice b. Challenge: OVA 50 mg	a. Sensitization: From Day 0 to Day 21 in weekly interval. b. Challenge: Day 28, 31, and 34.	16S rDNA gene sequencing	Compated to control, OVA ↑ Bacteroidetes and Proteobacteria and ↓ Firmicutes, meanwhile FAJ had opposite effect compared to OVA on these microbes. At genus level, OVA ↓ *Lactobacillus*, *Alistipes unclassified_f_Lachnospiraceae*, and *Lachnospiraceae_NK4A136_group* and ↑ *Alloprevotella* meanwhile FAJ had opposite effect of OVA on these genus.
Ma et al. (2025a)	Relationship between maternal dietary habit and food allergy in offspring	a. Serum b. Feces c. Jejunum	After 34 days	a. Maternal: HFD, FAJ + HFD; SD b. Offspring: HFD, HFD-A, FAJ + HFD-A; SD	Sensitization and challenge: Oral	a. Sensitization of offspring mice: OVA 1 mg + cholera toxin 10 μg b. Challenge of offspring mice: OVA 50 mg c. For maternal mice: 60% fat in HFD treatment. FAJ 0.15 mL/10 g mice	a. Sensitization: From Day 0 to Day 21 in weekly interval. b. Challenge: Day 28, 31, and 34.	16S rDNA gene sequencing	OVA ↓ Desulfobacterota, Firmicutes/Bacteroidota ratio and ↑ Proteobacteria and FAJ ↑ Proteobacteria and Firmicutes/Bacteroidota Maternal application of FAJ ↓ *Odaribactor*, *Parasuterella* and *Muribaculum* of sensitized offspring.

OVA, Ovalbumin; OVA/c, Ovalbumin + 10% cocoa diet; SF, Standard food; LAVA, Low Avenanthramide; HAVA, High Avenanthramide; APO, Apoptozole; PBS, Phosphate buffer saline; Lut, Luteolin; Myr, Myricetin; Hyp, Hyperoside; U-LG, Ultrasound treaded BLG; LG-LUT, Mixed BLG+ Luteolin; U-LG-LUT, Ultrasound treated LG-LUT; N-LG, Non-treated BLG; LVA, Low viscosity sodium alginate; bwt, Body weight; DMSO, Dimethyl sulfoxide; RA-low, Low concentration of rosmarinic acid; RA-Mid, Mild concentration of rosmarinic acid; RA-high, High concentration of rosmarinic acid; CT, cetirizine hydrochloride; NA, Not available; ↓, Decrease; ↓↓, Absent; ↑, Increase; ↑↑, Present.

#### 3.3.2 Soyabean and shrimp allergies

Although soyabean’s effect on microbes were not clearly mentioned, polyphenols such as luteolin reduced *Prevotella* and increased *Olsenella* (Liang et al., 2024) and. Unlike in ova allergy, 𝛽-conglycinin or green tea polyphenol (GTP) did not affect the *Akkermansia*, *Lachnospiraceae_NK4A136_group*, and Muribaculaceae. However, GTP promoted the *Bacteroides* and *Parabacteroides* (Zhou et al., 2023) (Table 2). The shrimp allergen reduced chitinophagaceae, rhizobiaceae, and increased burkholdariaceae, caulobactereaceae and sphingomonadaceae while the polyphenol extract had opposite effect compared to the allergen on these microbes (Feng et al., 2024) (Table 3).

**Table 3:** Main findings of soyabean and shrimp allergy studies.

Ref	Objectives	Tissue/cells/Sample	Collection time	Treatment; control	Administration route	Treatment dose	Treatment duration	Sequencing type	↑↓ Microbiome
Liang et al. (2024)	Effect of luteolin on intestinal health of piglets fed with soyabean meal based diet.	a. Blood b. Duodenum c. Jejunum d. Ileum. e. Digesta from colon	a. After day 31.	Luteolin diet (+ve control diet + 0.5% luteolin); casein, skimmed milk powder + fish meal (−ve control diet); soyabean meal (+ve control diet)	With feed	As per National Research Council’s recoomendation on nutritional requirement of swine	31 days	bacterial 16S rRNA gene amplicon sequencing	At phylum level, luteolin ↑ Actinobacteria, at genus level, luteolin ↓ *Prevotella* and ↑ *Olsenella*.
Zhou et al. (2023)	Anti-allergic effect and mechanism of GTP on BCN induced anaphylaxis	a. Blood b. Spleen c. Jejunum d. Digesta from cecum	Blood on day 15, 30, 45, and 60. All other samples on day 60	GTP; BCN (+ve), PBS (−ve)	PBS and BCN + PBS solution gavage. GTPs via water drinks	a. Control group: PBS 0.2 mL. b. BCN group: 0.2 mL BCN + 5 mg/mL PBS solution c. GTP group: 0.2 mL BCN + 5 mg/mL PBS solution + GTP 1% of rat’s drinks	PBS and BCN + PBS daily. GTP daily from day 0 to day 60.	16S rRNA gene sequencing	At phylum level, GTP ↑ Bacteroidetes and ↓ Firmicutes. At genus level, GTP ↑ *Bacteroides* and *Parabacteroides* compared to control and sensittization group. No difference among three groups for *Akkermansia*, *Lachnospiraceae_NK4A136_group*, *Lachnoclostridium*, Muribaculaceae, *Phascolarctobacterium*, and *Christensenellaceae_R-7_group*. No difference between GTP and sensitization gorup for *Ruminococcaceae_UCG-005*. At species level, compared to BCN, GTPs ↑ *Bacteroides uniformis*, *Bacteroides dorei*, *Parabacteroides goldsteinii*, and *Parabacteroides distasonis*.
Feng et al. (2024)	Anti-allergic properties of SBF	a. Blood b. Jejunum c. Feces	Serum and organ on days 15, 29, and 43.	SBF; TM (+ve), PBS (−ve)	SBF and PBS by intragastric gavage	a. Sensitization: TM and SBF group: 200 μg TM absorbed in 1 mg/mL IFA. SBF group: 200 μl of PBS conatining SBF (100 mg/kg bwt). PBS group: 200 μL of PBS b. Challenge: 1200 μg TM	a. Sensitization: Days 1, 7, 14, 21 TM b. Challenge: Day 43 c. SBF from day 14 to 42 daily	16S rDNA gene sequencing	At family level, compared to control group TM ↓ Chitinophagaceae, Rhizobiaceae and ↑ Burkholderiaceae, Caulobateraceae, and Sphingomonadaceae. The SBF modulated the declined Rhizobiaceae and Chitinophagaceae. The SBF ↑ Chitnophilidae and ↓ Burkholderiaceae, Shpingomonadaceae, Pneumatobacteriaceae.

GTP, Green tea polyphenol; BCN, 𝛽-conglycinin; PBS, Phosphate buffer saline; SBF, Sea buckthorn flavonoids extract; TM, Tropomysin; ↓, Decrease; ↑, Increase.

#### 3.3.3 Milk allergies

Milk allergy reduce firmicutes and increases bactereodota (Wang et al., 2022; Wang et al., 2024). The allergy also increases *Staphylococcus*, Campilobacterota and reduces *Lactobacillus*, *Alistipes*, *Odaribactor*, and *Bacteroides* (Wang et al., 2024). Use of various polyphenols such as flavonoids, luteolin, ferulic acids increases bifidobactereaceae, lactobacillaceae, *Faecalibacterium*, and *Agathobactor*. The polyphenol use reduced the staphylococceae, corynebactereaceae, and *Ramboustia* (Table 4).

**Table 4:** Main findings of milk allergy studies.

Ref	Objectives	Tissue/cells/sample	Collection time	Treatment; control	Administration route	Treatment dose	Treatment duration	Sequencing type	↑↓ Microbiome
Liu et al. (2021)	Structure of covalnet conjugates of bovine BLG and flanvonoids and their effect on allergenicity and human intestinal microbiota	Feces from non-allergic individuals	After 48 h of fermentation	Conjugates (BLG_Lut, BLG_Myr, BLG_Hyp); BLG	Incubation of conjugates with feces	All conjugates and BLG 10 mg	48 h of fermentation	16S rRNA gene sequencing	Conjugates ↑ Bacteroidota and ↓ Fusobacteroita compared to BLG. At family level, the conjugates ↑ Prevotellaceae, Lachnospiraceae, and Ruminococcaceae and *↓* Peptostreptococcaceae and Selenomomadaceae compared to BLG. At genus level, the conjugates ↑ *Prevotella*, *Faecalibacterium*, *Agathobacter* and ↓ *Romboutsia* and *Megasphera* compared to that of BLG.
Wang et al. (2022)	Effect of ultrasound on non-covalent interaction of BLG and luteolin and relation between allergenicity and intestinal microbiota	Stool from healthy individuals	After 48 h of fermentation	U-LG-LUT, S-LG-LUT, LG-LUT, U-LG; N-LG	NA	In KU812 cell culture, a. Sensitization: with human serum IgE of milk for 24 h b. Stimulation: with treatments 50 μL/well (1 mg/mL) for 4 h	48 h of anaerobic sterile fermentation	16S rRNA gene sequencing	At phylum level, compared to N-LG, other treatment ↑ Firmicutes and Proteobacteria while ↓Bacteroidota. At family level, compared to control group, N-LG and U-LG ↑ Prevotellaceae and ↓ Selenomonadaceae and Bifidobacteriaceae. The U-LG-LUT and S-LG-LUT increased the Bifidobacteriaceae compared to N-LG and U-LG. At genus level, compared to control, all the LG included treatment ↓ *Megamonas* and *Bifidobacterium* and ↑ *Prevotella*. Compared to N-LG, the S-LG-LUT and U-LG-LUT ↑*Bifidobacterium*.
Wang et al. (2024)	Anti-allergic effect of the ferulic acid and glucose combination on BLG	a. Serum b. Feces	After day 40	A-BLG, BLG-FA, BLG-Glu, BLG-FA-Glu; BLG (+ve)	Sensitization and Challenge: Oral	a. Sensitization: 5 mg b. Challenge: 20 mg	a. Sensitizaiton: from day 0 to day 35 in weekly interval b. Challenge: Day 40	16S rRNA gene sequencing	At phylum level, compared to BLG, all other treatments ↓ Firmicutes and Actinobacteria and ↑ Bacteriodota and Campilobacterota. At family level, campared to other treatments, BLG ↓ Lactobacillaceae, Rikenellaceae, Lachnospiraceae, Marinifilaceae, and Bacteriodaceae and ↑ Corynebacteriaceae and Staphylococcaceae. BLG-FA ↑ Lactobacillaceae and ↓ Staphylococcaceae and Corynobacteriaceae. The BLG-Glu and BLG-FA-Glu ↑ Lachnospiraceae and ↓ Corynebacteriaceae. At Genus level, BLG ↓ *Lactobacillus*, *Alistipes*, *Odaribactor*, and *Bacteroides* and ↑ *Staphylococcus* and *Corynebacterium*.

BCN, 𝛽-conglycinin; BLG, 𝛽-lactoglobulin; Lut, Luteolin; Myr, Myricetin; Hyp, Hyperoside; U-LG, Ultrasound treaded BLG; LG-LUT, Mixed BLG+ Luteolin; U-LG-LUT, Ultrasound treated LG-LUT; N-LG, Non-treated BLG; A-BLG, Alkaline 𝛽-lactoglobulin; BLG-FA, 𝛽-lactoglobulin + ferulic acid; BLG-Glu, 𝛽-lactoglobulin + glucose; BLG-FA-Glu, 𝛽-lactoglobulin + ferulic acid + glucose; NA, Not available; ↓, Decrease; ↑, Increase.

## 4 Discussion and future perspectives

In general, food allergy is related to reduction *of Lactobacillus*, *Alistipes*, *Odaribactor*, *Akkermansia*, *Bacteroides*, and *Lachnospiraceae_NK4A136_group* and an increase of *Prevotella*, *Alloprevotella*, *Faecalibaculum*, *Helicobactor*, *Blautia*, *Clostridium*, and *Staphylococcus* (Tables 2–4). Previous studies also found that food allergy is related to reduced *Bacteroides*, *Alistipes*, *Lachnospriaceae_NK4A136_group*, *Akkermansia*, and *Lactobacillus* and abundance of *Prevotella*, *Helicobacter* and *Clostridium* (Chen et al., 2016; Gu et al., 2022; Tanaka et al., 2024; Liu et al., 2019; Huang et al., 2025; Hara et al., 2024; Chang et al., 2018; E et al., 2024; Xu et al., 2025; Qiao et al., 2024).

Very few common microbial species were identified across different food allergy studies. Moreover, the effect of polyphenols on food allergies varied according to the type of polyphenol used and type of food allergies. For example, cocoa (Camps-Bossacoma et al., 2017), flavonoids (Liu et al., 2021), and Luteolin (Wang et al., 2022) increased *Prevotella* compared to allergen alone in milk and egg allergies. However, the *Prevotella* was reduced by the Luteolin compared to that of soybean allergen (Liang et al., 2024). This implies that the microbiome’s role also varies based on food allergy type (Goldberg et al., 2020; De Filippis et al., 2021). Similar, variation was also found in previous studies where *Prevotella copri* was increased in milk allergy while it was decreased in peanut allergy (Goldberg et al., 2020). Futhermore, *Blautia* was increased by cyanidin-3-O-glucoside (C3G) (Li et al., 2022) and Avenanthramide’s (AVA) (Liu et al., 2023) but it was reduced by cocoa diet (Camps-Bossacoma et al., 2017) compared to that of allergens alone. Furthermore, the C3G (Li et al., 2022) increased *Lactobacillus alistipes* and while cocoa diet (Camps-Bossacoma et al., 2017) increased the *Lactobacillus reuteri* compared to allergen alone. At phylum level, cocoa diet (Camps-Bossacoma et al., 2017), green tea polyphenol (GTP) (Zhou et al., 2023), or rosmarinic acid (Yang et al., 2023; Zhou et al., 2023) decreased firmicutes, but C3G (Li et al., 2022; Zhou et al., 2023) increased the firmicutes in comparison to the allergens. Other studies investigating the effect of different polyphenols in microbes have also reported the different in effect of various polyphenols on the same genus/species of microbes (Loo et al., 2020; Mithul Aravind et al., 2021). Thus, more studies are needed on the effect of polyphenols on a specific types of food allergies via microbiome modulation in order to identify signature microbiome modulation pattern of the specific types of allergy before determining the pattern for food allergies in general.

Besides the types of polyphenols or food allergens, the taxonomic resolution of the microbiome in a study may also affect the results. For example, effects of both the C3G and cocoa diet were measured at species level, i.e., they both increased the *Lactobacillus alistipes* and *Lactobacillus reuteri*, respectively. However, the cocoa diet increased *Lactobacillus reuteri* but decreased *Ruminococcus flavefaciens* compared to allergen. Both of these bacterial species are firmicutes, but represent different classes, orders, or families (Camps-Bossacoma et al., 2017; Zhou et al., 2023). Similarly, the C3G (Li et al., 2022) or AVA (Liu et al., 2023) increased *Blautia*, but cocoa diet (Camps-Bossacoma et al., 2017), reduced *Blautia producta* compared to the allergen treatment. Furthermore, cocoa diet increased the *Clostridium metallovans*, but it caused a disappearance of the *Clostridium perfringens* compared to standard food. Similarly, in other studies, *Clostridium senso stricto1* found in healthy children while *Clostridium innnocuum* were higher in wheat allergic children (Kanchongkittiphon et al., 2024) Not only species but also strains of a species vary in their presence and function (Mennini et al., 2021). These results indicate that it is important to study the higher level of taxonomic resolution of the microbiome in order to accurately determine the effect of polyphenols in food allergy via microbiome modulation.

## 5 Limitation

This study included the articles published in English. Thus, it may cause omission of important articles in other languages. The included studies also had diverse polyphenol forms and animal models (rats, mice, and piglets). These cause variation in the results, making it hard to find common microbiome signature and their modulation pattern by polyphenol. All included studies used 16S rRNA gene sequencing to investigate the microbiome changes due to the polyphenols. The 16S rRNA sequencing is not rigorous enough to study at species or strain level of microbes. Moreover, some of the studies only reported the results at phylum and genus level, which increased the variability of the microbiome results. Most of the other food allergies and gut microbiome studies also reported microbiome diversity and functional prediction using 16S rRNA sequencing techniques (Chen et al., 2016; Goldberg et al., 2020; Gu et al., 2022; Kourosh et al., 2018; Bunyavanich and Berin, 2019; Tanaka et al., 2024; Mennini et al., 2021; Fazlollahi et al., 2019; Tulyeu et al., 2019). Very few studies have used combined approaches such as the 16S rRNA gene sequencing and metabolomics (Xu et al., 2022) or shutgun metagenomics (De Filippis et al., 2021) to determine microbial signature and their potential functional in various food allergies. Furthermore, out of other important food allergies, only four types were covered by the included studies (egg, milk, shrimp, and soybean). Other important food allergies such as allergies related to peanut, wheat, and tree nuts are yet to be studied in terms of polyphenol’s effect on these allergies via microbiome modification. Thus, finding in this study is limited to modulation of polyphenols on egg, milk, soyabean and shrimp allergy. For modulatory effect of polyphenol on the other important food allergies including peanut, wheat, and nuts, further studies are necessary in the future.

## 6 Conclusion

Higher level of variation in polyphenol used and animal model used along with lower taxonomic resolution of microbiome in the included studies in this review led to lack of common microbiome modulation pattern of polyphenols in the reduction of food allergy. High-resolution taxonomic level investigation (Jovel et al., 2016) or microbiomes-and-metabolomics approach (Xu et al., 2022) have been proven effective in getting the signature gut microbiome in food allergy studies. Given that 16S rRNA sequencing technique would not provide the species or strain level resolution which is critical for identification of signature microbiome and their functional potential in food allergy. Moreover, shotgun sequencing approach provide higher taxonomic resolution and opportunity to direct assessment of functional potential of the microbiomes (Jovel et al., 2016). Thus, use of shotgun metagenomics combined with metabolomics could provide reliable food allergy microbiome signature and their potential function as well as reliable measure of polyphenol’s effect on food allergy via microbiome modulation.

## Data Availability

The original contributions presented in the study are included in the article/Supplementary material, further inquiries can be directed to the corresponding author/s.
