# Supplementary material for: A systematic review: polyphenol’s effect on food allergy via microbiome modulation
Source: Front Microbiol. 2025 Nov 18;16:1673472. doi: 10.3389/fmicb.2025.1673472 (PMC12669167; doi:10.3389/fmicb.2025.1673472)
Supplement: Supplementary file 1 [file Data_Sheet_1.pdf]

Supplementary Material

1 Supplementary Tables

Supplementary Table 1. Risk of bias assessment of *in vitro* study based on modified CONSORT checklist.

Author year	Abstract 1	Introduction		Methods			Results	Discussion	Other information
		2a	2b	3	4	10	11	12	13
		Background & Rationale	Objectives (and/or hypothesis)	Intervention	Outcomes	Statistical methods	Outcomes and estimations	Limitations	Funding
Liu et al. 2021 (1)	Yes	Yes	Yes (No)	No	Yes	No	Yes	Yes	Yes
Wang et al. 2022 (2)	Yes	Yes	Yes (No)	No	Yes	Yes	Yes	Yes	Yes

Supplementary Table 2. Risk of bias assessment of *in vivo* studies based on SYRCLE's risk of bias tool.

Reference	Was the allocation sequence adequately generated and applied? (1)	Were the groups similar at baseline or adjusted for confounders? (2)	Was the allocation adequately concealed? (3)	Are the animals randomly housed during the experiment? (4)	Were the caregivers/ investigators adequately blinded during the course of the experiment? (5)	Were animals selected at random during outcome assessment (6)	Was the outcome assessor adequately blinded? (7)	Were incomplete outcome data adequately addressed? (8)	Is the study free of selective outcome reporting? (9)	Was the study apparently free of other problems that could cause a high risk of bias? (10)
Camps-Bossacoma et al. 2017 (3)	Unclear	Yes	Unclear	Unclear	Unclear	Unclear	Unclear	Yes	Yes	Yes
Li et al. 2022 (4)	No	Yes	Unclear	Unclear	Unclear	Unclear	Unclear	Unclear	Yes	Yes
Liang et al. 2024 (5)	No	Yes	Unclear	Unclear	Unclear	Unclear	Unclear	Yes	Yes	Yes
Liu et al. 2023 (6)	Unclear	Yes	Unclear	Unclear	Unclear	Unclear	Unclear	Yes	Yes	Yes
Yang et al. 2023 (7)	Unclear	Yes	Unclear	Unclear	Unclear	Unclear	Unclear	Unclear	Yes	Yes
Zhou et al. 2023 (8)	Unclear	Yes	Unclear	Unclear	Unclear	Unclear	Unclear	Yes	Yes	Yes

References

(1) Liu J, Wang Y, Tu Z, Chen W, Yuan T. Bovine β-Lactoglobulin Covalent Modification by Flavonoids: Effect on the Allergenicity and Human Intestinal Microbiota. *J Agric Food Chem* (2021) **69**:6820. doi: 10.1021/acs.jafc.1c02482.

(2) Wang T, Chen W, Shao Y, Liu J, Tu Z. Ultrasound Improved the Non-Covalent Interaction of β-Lactoglobulin with Luteolin: Regulating Human Intestinal Microbiota and Conformational Epitopes Reduced Allergy Risks. *Foods* (2022) **11**. doi: 10.3390/foods11070988.

(3) Camps-Bossacoma M, Pérez-Cano FJ, Franch À, Castell M. Gut Microbiota in a Rat Oral Sensitization Model: Effect of a Cocoa-Enriched Diet. *Oxidative Medicine and Cellular Longevity* (2017) **2017**. doi: 10.1155/2017/7417505.

(4) Li J, Zou C, Liu Y. Amelioration of Ovalbumin-Induced Food Allergy in Mice by Targeted Rectal and Colonic Delivery of Cyanidin-3-O-Glucoside. *Foods* (2022) **11**. doi: 10.3390/foods11111542.

(5) Liang X, Zheng S, Zhou Y, Li J, Zhang Z. Luteolin, a natural flavonoid, exhibits a protective effect on intestinal injury induced by soybean meal in early-weaned piglets. *Journal of Animal Science* (2024) **102**. doi: 10.1093/jas/skae214.

(6) Liu P, Zhang M, Liu T, Mo R, Wang H, Zhang G, et al. Avenanthramide Improves Colonic Damage Induced by Food Allergies in Mice through Altering Gut Microbiota and Regulating Hsp70-NF-κB Signaling. *Nutrients* (2023) **15**. doi: 10.3390/nu15040992.

(7) Yang Q, Jia B, Shang J, Wang X, Xu L, Liu X, et al. Effects of rosmarinic acid on immune response and intestinal microbiota in ovalbumin-induced intestinal allergy mice. *J Sci Food Agric* (2023) **104**:3002. doi: 10.1002/jsfa.13192.

(8) Zhou Y, Zheng S, Yang S, Li J, Yang K, Han J, et al. Green tea polyphenols alleviate β-conglycinin-induced anaphylaxis by modulating gut microbiota in rats. *Food Bioscience* (2023) **56**. doi: 10.1016/j.fbio.2023.103339.
